# Rare abdominal wall clear cell carcinoma in endometriosis: case report and literature review of diagnostic and therapeutic considerations

**DOI:** 10.1186/s12957-025-03974-0

**Published:** 2025-10-15

**Authors:** Davut Dayan, Lisa Wisniewski, Andrea Formentini, Marko Kornmann, Sarah Bette, Florian Ebner, Wolfgang Janni, Stefan Lukac, Lea Traub, Fabienne Schochter

**Affiliations:** 1 Department of Obstetrics and Gynaecology, University Hospital Ulm, Ulm, Germany; 2 Department of Visceral Surgery, University Hospital Ulm, Ulm, Germany; 3 Department of Obstetrics and Gynaecology, Alb-Donau Klinikum Ehingen, Ehingen (Donau), Germany; 4 Institute of Pathology, University Hospital Ulm, 89081 Ulm, Germany

**Keywords:** Endosalpingiosis, Endometriosis, Clear cell carcinoma of the abdominal wall, Recurrent abdominal wall tumors, Management of abdominal wall reconstruction

## Abstract

**Background:**

Endometriosis of the abdominal wall is exceptionally rare, with malignant transformation to clear cell carcinoma being of even greater rarity. In this paper, the importance of accurate diagnosis and appropriate treatment is emphasized, including the possibility of mesh reconstruction of the abdominal wall.

**Case presentation:**

A 53-year-old woman presented with a rapidly enlarging abdominal wall tumor, 19 years after undergoing a caesarean section. Prior to her referral to our facility, a peripheral hospital had already performed tumor resection with mesh-based abdominal wall reconstruction. Tumor histology had revealed endosalpingiosis without evidence of malignancy. However, a year later, the patient exhibited a relapse accompanied by renewed rapid growth. Both punch and open biopsies revealed endosalpingiosis. Due to its rapid growth, a total resection of the tumor was performed. This necessitated the removal of a significant portion of the abdominal wall musculature and fascia. The abdominal wall was then reconstructed using a two-layer mesh. Final histological examination revealed a clear cell carcinoma that had arisen from endometriosis. Following the complete healing, the patient received adjuvant platinum-based chemotherapy and radiation to the inguinal area.

**Conclusion:**

Clear cell carcinoma of the abdominal wall arising from endometriosis is a rare and highly aggressive form of malignant transformation with a high rate of recurrence and metastasis. In rapidly growing abdominal wall tumors following gynaecological surgery, the objective should be radical resection. In addition to this, the selection of appropriate reconstruction procedures is crucial to achieving optimal oncological and functional results. Examples of such reconstruction procedures include a two-layer mesh reconstruction.

This case aims to raise awareness of this rare and highly aggressive malignancy and to discuss diagnostic challenges and therapeutic strategies, including complex abdominal wall reconstruction.

## Introduction

Endometriosis is a common, benign, chronic inflammatory disease affecting approximately 10% of women of reproductive age. It is defined by the presence of endometrial-like epithelium and/or stroma outside the uterine cavity [6, 11, 35]. A closely related benign condition of Müllerian origin is endosalpingiosis, which shares histopathological similarities with endometriosis. It is characterised by ectopic proliferation of ciliated tubal-type epithelium or endometrial glands and stroma [10, 25], and is reported as a concurrent finding in approximately 4.4–67% of endometriosis cases [16, 24, 27, 39]. Previous pelvic surgery is recognised as a potential risk factor for both entities [28]. Tumor scar endometriosis following abdominal surgery occurs in approximately 0.03–1.08% of cases [37], with the occurrence of malignant transformation in less than 1% of diagnosed cases [1, 7, 30]. While transformation most commonly affects the ovaries, extragonadal sites such as the colon, rectovaginal septum and vaginal wall may also be involved.

In 1925, Sampson defined three diagnostic criteria for the malignant transformation of endometriosis:

1. clear evidence of endometriotic lesions adjacent to the tumor,
2. a tumor morphology consistent with endometrial origin, and
3. exclusion of another primary malignancy [9, 38, 42].

In 1953, Scott proposed a fourth criterion: histological evidence of transition between benign endometriosis and malignant epithelium [44].

The most common histological subtypes of extragonadal malignancies arising from endometriosis are endometrioid carcinoma, clear cell carcinoma, and sarcomas [4, 5, 38].

Clear cell carcinomas most frequently develop in the ovary, but may also arise in the endometrium or cervix. Despite differing anatomical locations, they share similar histological and biological features. Notably, ovarian clear cell carcinomas exhibit considerable resemblance to renal clear cell carcinomas.

Endometriosis is a well-established risk factor for clear cell ovarian carcinoma, increasing the risk approximately threefold [22]. It is estimated that up to 50% of ovarian clear cell carcinomas are associated with endometriosis, particularly in women with long-standing disease [4, 14]. 

Malignant transformation of abdominal wall endometriosis typically occurs gradually and may manifest years after previous surgical procedures. The median age at diagnosis is between 40 and 50 years [17, 37, 47].

The central objective of this report is to underscore the importance of early diagnosis and prompt, adequate surgical intervention in rare cases of clear cell carcinoma of the abdominal wall arising in the context of endometriosis or endosalpingiosis, particularly following gynaecological surgery.

In addition, we highlight the role of two-layer mesh reconstruction in managing extensive abdominal wall defects following tumor resection.

### Tumor case presentation

In our case a 53-year-old caucasian postmenopausal woman was referred to our university hospital from an external clinic with a large, progressive and symptomatic recurrence of an abdominal wall tumor.

Prior to her first delivery, she had complained of dysmenorrhoea, which had been rated at 6/10 on the VAS (visual analogue scale) and had occurred one day before and on the first day of her period. This had only required occasional pain medication. After her pregnancies, these symptoms had disappeared completely. She had not experienced any other symptoms that could indicate endometriosis and had not yet been diagnosed with the condition. She had her second child 19 years ago by caesarean section due to failure to progress during labour. The patient has no history of illness and has not undergone any other operations apart from the caesarean section. The family history is unremarkable. She first noticed swelling in the scar approximately one and a half years ago. Due to the tumor’s rapid progression and associated pain, it was removed from the lower abdomen one year ago. A 20 × 25 cm Parietene^®^ composite mesh was implanted to stabilise the abdominal wall. Histological examination revealed endosalpingiosis with no evidence of malignancy. No further treatment was recommended. Following the procedure, the patient was symptom-free for about six months. However, she subsequently noticed a palpable mass in her lower abdomen, which grew larger and became more sensitive to pressure over time. Despite ongoing opioid analgesic treatment, she continued to experience severe pain, rating it as high as 8/10 on the VAS.

A physical examination revealed an obese abdominal wall with a healed transverse laparotomy scar, no skin changes, and no palpable lymph nodes. Palpation revealed a 20 cm tumor in the lower abdomen that was both firm and painful, extending just below the skin.

**Fig. 1:**
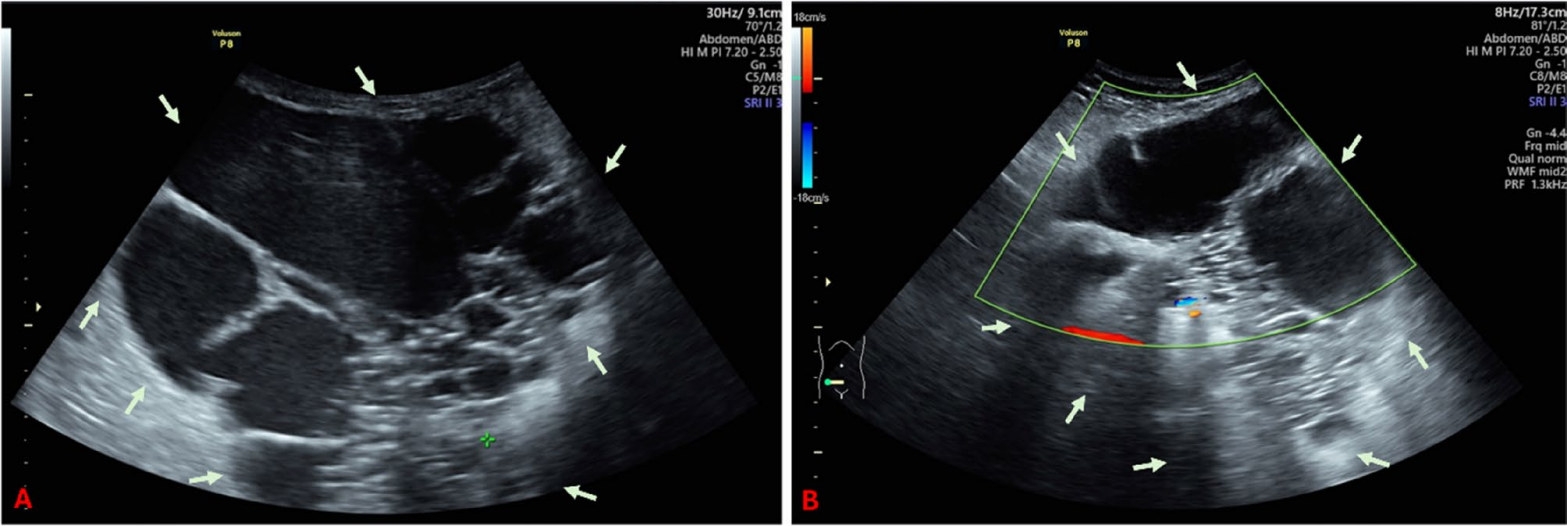
A: Transabdominal ultrasound image of the predominantly cystic abdominal wall tumor. B: Doppler sonographic image of the blood flow in the hyperechoic area of the tumor. The lesion is marked with arrows

**Fig. 2:**
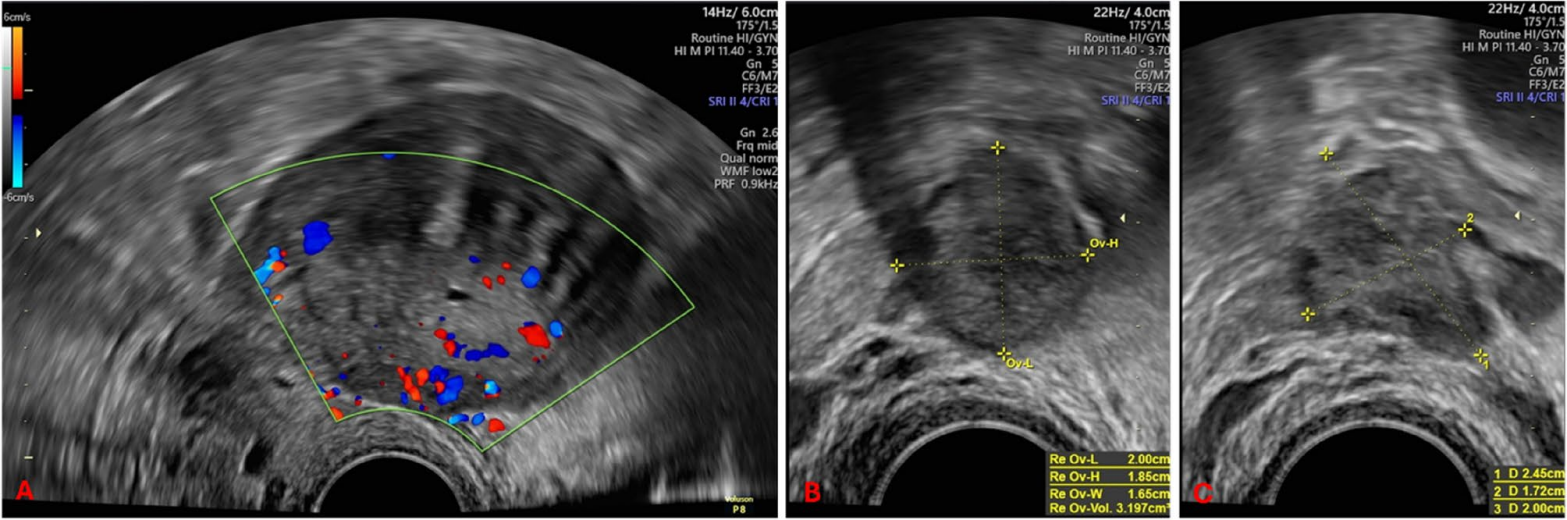
Transvaginal ultrasound imaging of the internal genitalia. A: longitudinal section of the uterus, with suspected uterine adenomyosis; B: normal right ovary; C: normal left ovary

The gynaecological examination was unsignificant. Sonography in the lower abdomen revealed the presence of multiple echo-free, multi-chamber cysts, devoid of any suspicious perfusion signs (see Fig. 1). Transvaginal ultrasonography does not yield any further pathological findings except a strong suspicion of uterine adenomyosis (see Fig. 2).

**Fig. 3:**
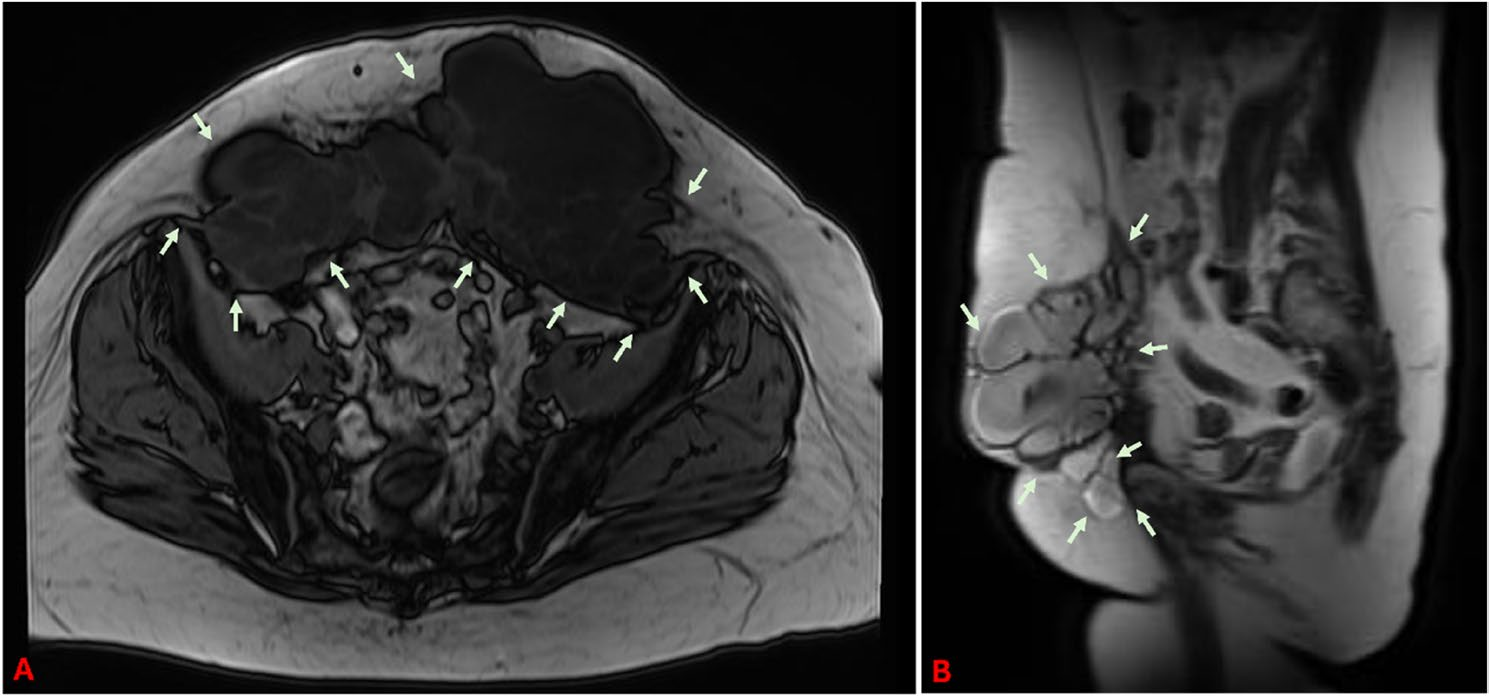
Magnetic resonance imaging (MRI) of the abdominal wall tumor: A: Axial view; B: Sagittal view. We sincerely thank Prof. Dr. Beer, Medical Director of Radiology at Ulm University Hospital, and his team for kindly providing the images. The tumor is marked with arrows

Magnetic resonance imaging (MRI) showed a 21 × 16 cm cystic septated mass of the ventral abdominal wall, with a solid caudal portion and suprasymphysary contrast agent accumulation (6.4 cm). The lesion extended to the cutis and laterocaudally to the inguinal and femoral canal. This finding suggested an extensive local recurrence of endosalpingosis with infiltration of the right inguinal/femoral canal. The potential for malignancy could not be ruled out (see Fig. 3).

Ultrasound-guided cytological puncture yielded only cell-rich material containing leukocytes, macrophages, and cellular debris. Subsequent open tissue sample with histopathological examination revealed a regressive endometriotic cyst embedded in dense fibrotic scar tissue without any evidence of malignancy. At 17.4 (normal < 35 IU/ml), tumor marker CA 125 was within the normal range. However, given the persistent symptoms and the rapid tumor growth, a Multidisciplinary Tumor Board (MTB) concluded that tumor resection with subsequent abdominal wall reconstruction should be recommended. Due to the extensive tumor spread and the consequently limited surgical options, hysterectomy with bilateral salpingo-oophorectomy was offered.

Intraoperative examination revealed a multicystic tumor reaching from the peritoneum, infiltrating the skin in the lower abdomen, and from the peritoneum to the fascia in the middle and upper abdomen. The mass stretched from the mons pubis to just below the costal margin, entrapping the right artery and external iliac vein, as well as the femoral vessels. Furthermore, the tumor infiltrated the right groin, spreading to the thigh musculature and involving the roof of the bladder. With the exception of adenomyosis, no evidence of endometriosis could be found intraabdominally.

Utilising intraoperative ultrasonography and meticulously preserving non-infiltrated skin and subcutaneous tissue, the abdominal wall was resected from the mons pubis to the upper abdomen. The preparation of the tumor surrounding the pubic bone necessitated the utilisation of monopolar electrocautery. Concurrently, a hysterectomy was performed, accompanied by bilateral salpingo-oophorectomy and the resection of enlarged iliac lymph nodes. The infiltrated bladder apex was resected, and the bladder was sutured free of tension.

**Fig. 4:**
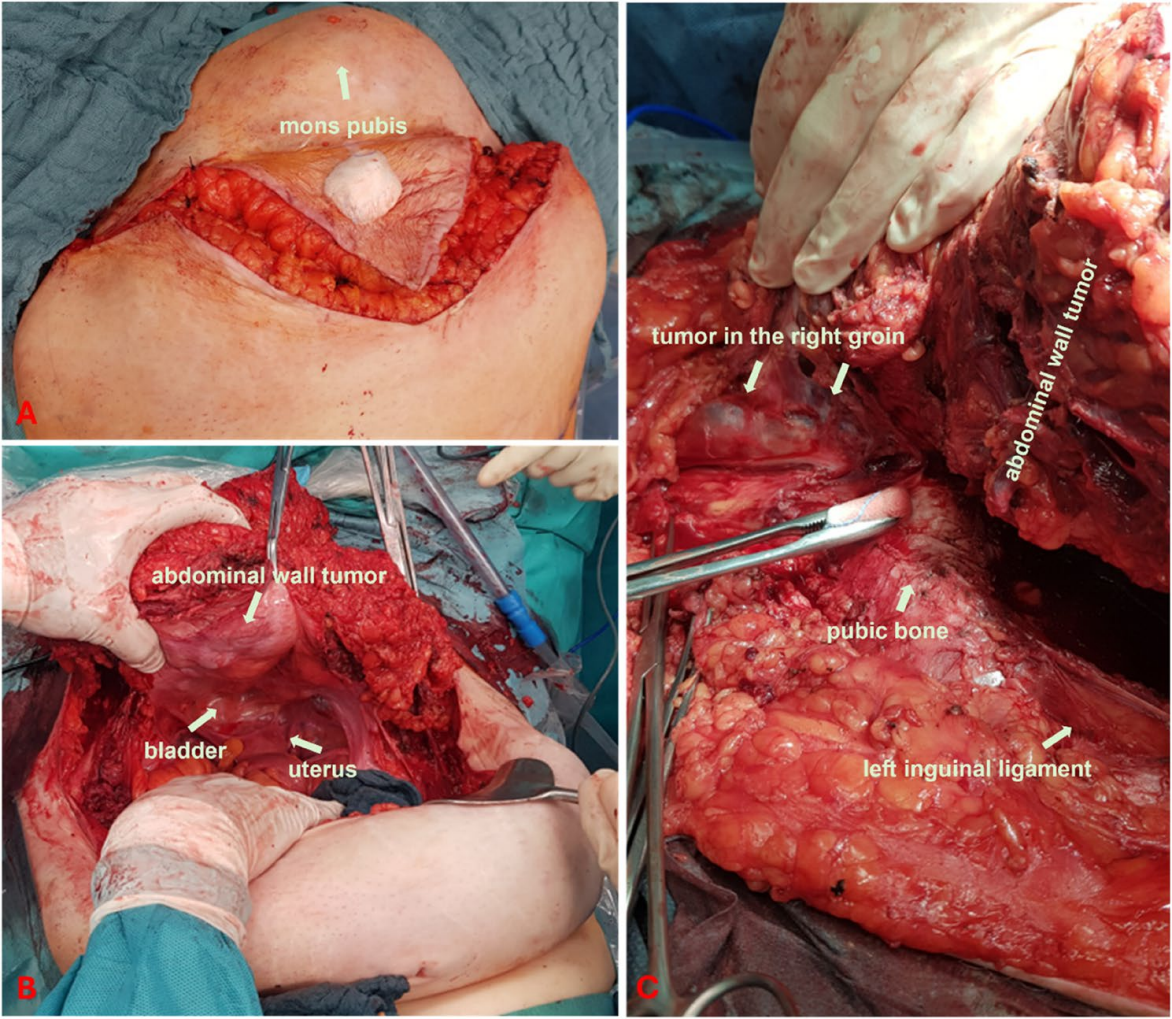
Intraoperative findings A: Exposure of the incision line B: Exposure of all layers of the infiltrated abdominal wall with cystic tumor foci C: Exposure of the tumor foci in the right thigh after detachment of the tumor from the pubic symphysis

**Fig. 5:**
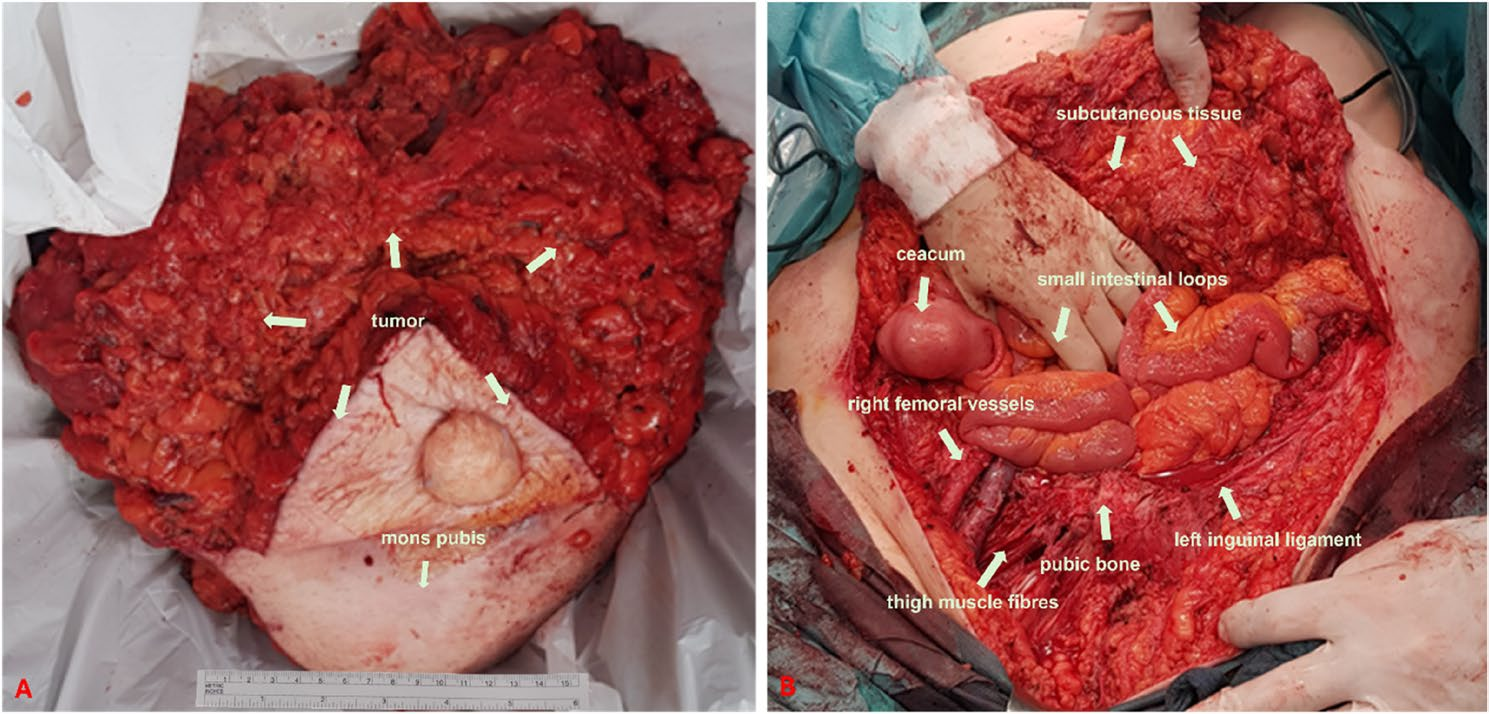
A: Description of the removed surgical specimen B: Description of the surgical site after removal of the tumor

The cystic tumor parts in the right thigh were completely resected after careful dissection for protection of nerves, vessels and non-involved muscle (see Figs. 4 and 5). Macroscopically, the extent of the surgical intervention was found to be tumor-free. The resected specimen was sent to pathology for frozen section examination.

**Fig. 6:**
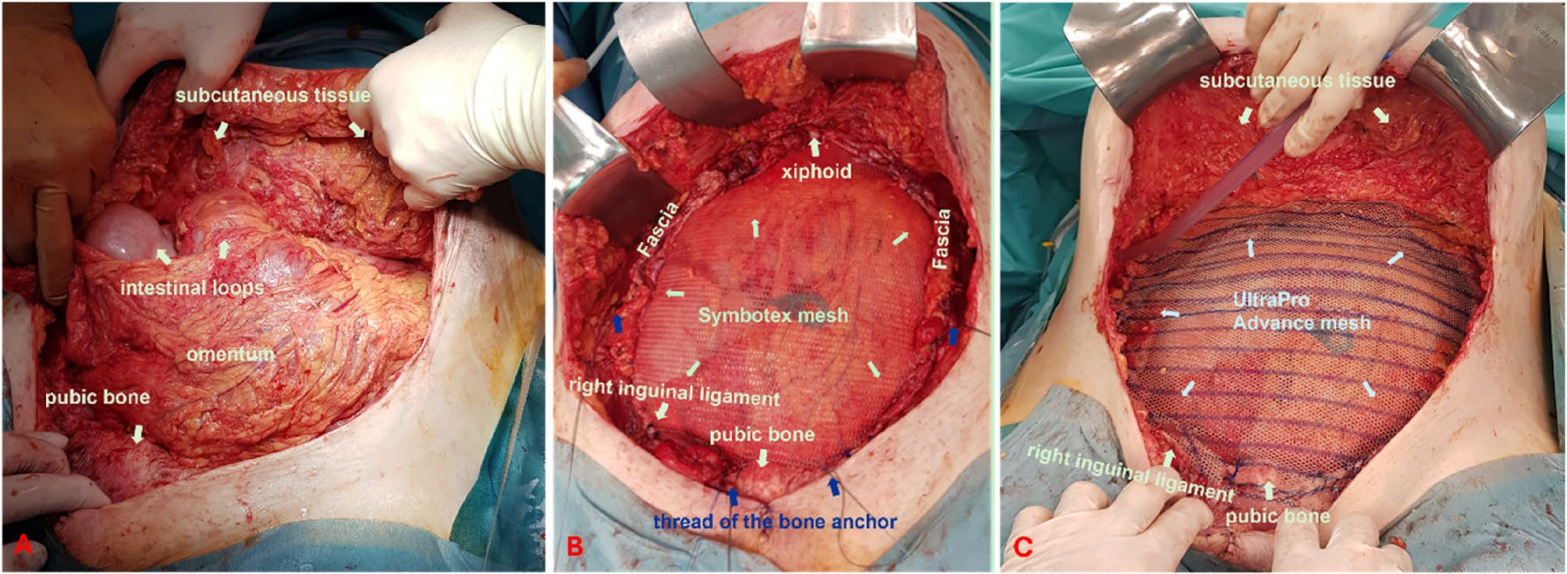
Intraoperative views of abdominal wall reconstruction: A: Omental flap positioned as a protective barrier between the mesh and the intestines. B: Inner layer reconstruction using Symbotex^®^ mesh, extending from the symphysis to the xiphoid process. The mesh was placed above the omentum and beneath the residual abdominal wall, and fixed superiorly to the remaining fascia, in the mid and lower abdomen to stable remnants of the abdominal wall, and inferiorly to the bone using prepositioned anchor sutures (blue arrows). C: Outer layer reconstruction using UltraPro^®^ Advance mesh, placed as a superficial layer over the Symbotex^®^ mesh. It was likewise fixed superiorly to the remaining fascia, and laterally and inferiorly to stable areas of the abdominal wall and the bone using anchor sutures. The resected right inguinal ligament was reconstructed using a two-layer UltraPro^®^ Advance mesh technique

**Fig. 7:**
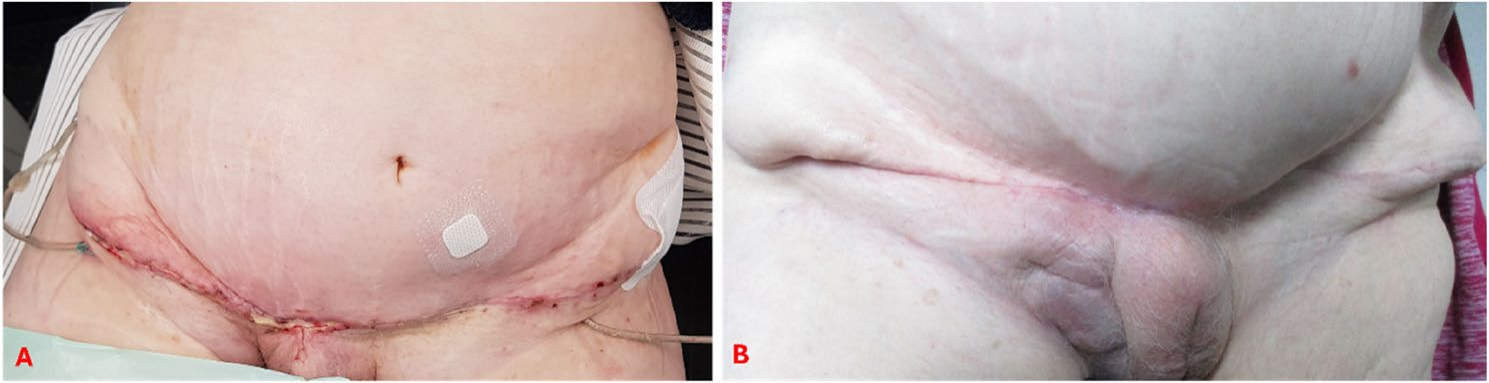
Appearance of the abdominal wall after reconstruction A: 3 weeks after surgery B: 1 year after surgery

The tumor frozen section examination resulted in a strong suspicion of endosalpingiosis, though no malignancy could be detected. Furthermore, the frozen section did not reveal any signs of lymph node infiltration. In the absence of any evidence of malignancy, further oncological measures, such as lymph node removal or omentectomy, were not pursued. Due to the extensive nature of the abdominal wall resection, primary wound closure was not feasible. Initially the abdominal wall was temporarily closed with a dressing system. Following the completion of preparatory procedures, the final abdominal wall closure was performed implanting a Symbotex^®^ mesh (42 × 32 cm) as the inner layer and an UltraPro^®^ Advance mesh (1 × 30 × 30 cm and 1 × 15 × 30 cm) as the outer layer to reconstruct the abdominal wall. Given the considerable restriction of further operability that would ensue from such extensive abdominal wall reconstruction, it was decided to perform prophylactic appendectomy and cholecystectomy, which were carried out successfully in the usual manner. Due to the infiltration of the abdominal wall, it was necessary to resect the entire lower abdominal wall down to the bone. To secure the mesh directly to the bone, bone anchors were required. These were prepositioned bilaterally at the pubic bone and the anterior superior iliac spines (see Fig. 6B, blue arrows). The greater omentum was then mobilised and fixed bilaterally to the residual abdominal wall to create an “omental seal” between the intestines and the mesh (see Fig. 6A). The Symbotex^®^ mesh was placed above the omentum and beneath the residual abdominal wall. It was fixed in the upper abdomen to the remaining fascia, in the mid-abdomen and lower abdomen to residual stable parts of the abdominal wall as well as inferiorly to the bone using the prepositioned anchor sutures. Subsequently, the UltraPro^®^ Advance mesh was placed superficially over the Symbotex^®^ mesh. It was also fixed in the upper abdomen to the remaining fascia, and laterally and in the lower abdomen to stable remnants of the abdominal wall and to the bone using the anchor sutures. Additional fixation was achieved by suturing it to the underlying Symbotex^®^ mesh. The resected right inguinal ligament was reconstructed using a two-layer UltraPro^®^ Advance mesh (see Fig. 6). Following the placement of surgical drains in all four quadrants of the abdominal wall, the surgical team proceeded to close the wound using a technique that involved the adaptation of the spared skin. During the surgery, the patient received prophylactic antibiotics cefuroxime and metronidazole, and following surgery Unacid^®^ antibiotic prophylaxis was administered. Delayed wound healing was observed, yet primary healing was eventually achieved (see Fig. 7).

**Fig. 8:**
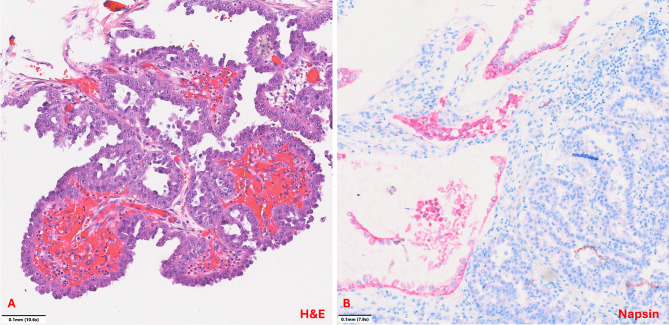
Histopathology demonstrating. Clear cell carcinoma in proximity to the Peritoneal wall. A: The clear cell carcinoma shows characteristic papillary architecture lined by cuboidal „hobnail“ cells with eospinophilic cytoplasm and enlarged atypical nuclei with prominent nucleoli. The mitotic count is low (H&E stain; 10,6x). B: Napsin positivity was observed in a subset of tumor cells (Napsin stain; 7,9x). We would like to thank Professor Gaisa, the Medical Director of Pathology at Ulm University Hospital, and her team for kindly providing these images

The final histopathological examination revealed a high-grade clear cell carcinoma (see Fig. 8) of the abdominal wall at the base of a seromucinous borderline tumor with infiltration of the mons pubis and thigh tissue. Micrometastatic involvement was detected in one of the fatty tissue lymph nodes, while the iliac lymph nodes remained tumor-free bilaterally. There were no signs of tumor manifestation in other abdominal organs. Additionally, the uterus and adnexa were found to be free of tumor cells, although pronounced uterine adenomyosis and isolated extrauterine endometriosis lesions were detected. The TNM classification was pT4, pN1mi (1/3), L0, V0, Pn0, RX. Immunohistochemical analysis revealed a weak expression of estrogen receptors in 10% of cells (IRS 1), and an absence of progesterone receptor expression. Additionally, a Ki-67 proliferation index of 20–30% was observed. Furthermore, the tumor was found to be L1CAM negative, exhibited a p53 wild type, and did not demonstrate microsatellite instability.

**Fig. 9:**
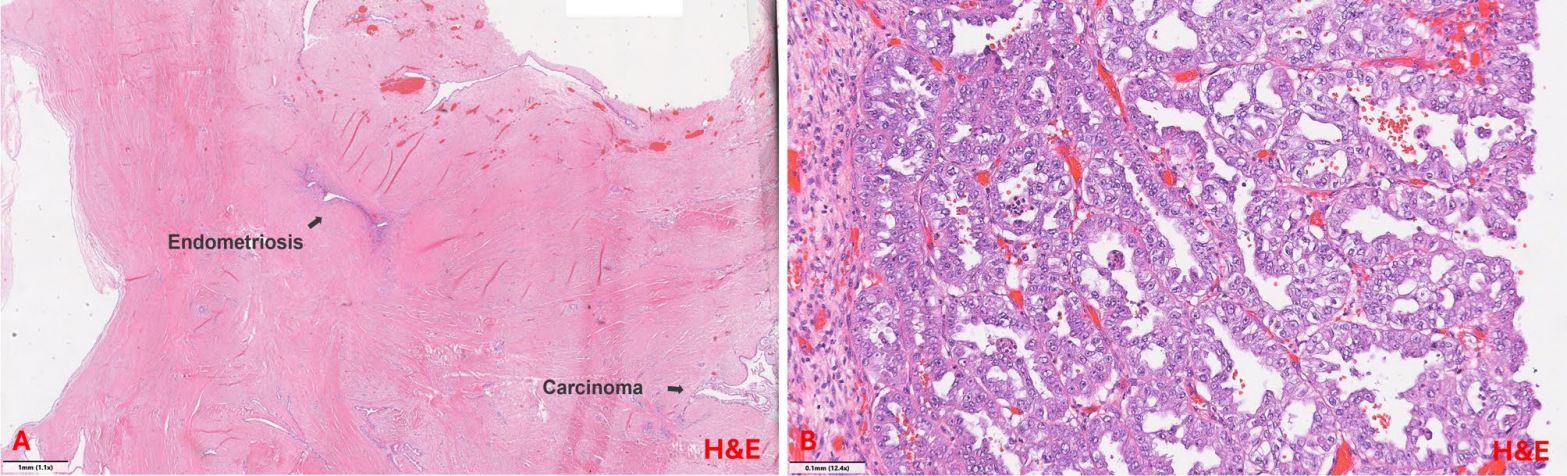
Histopathology demonstrating A: Endometriosis in proximity to cystic carcinoma formations (H&E stain; 1,1x). B: Clear cell carcinoma. The clear cell carcinoma shows infiltrative growth with a combination of tubulocystic and papillary architecture lined by cuboidal „hobnail“ cells with clear as well as eospinophilic cytoplasm. The cells only show minimal stratification. Nuclei are monomorphic and enlarged with prominent nucleoli and a low mitotic count (H&E stain; 12,6x). We would like to thank Professor Gaisa, the Medical Director of Pathology at Ulm University Hospital, and her team for kindly providing these images. The lesion is marked with arrows

Given the absence of tumor infiltration in the uterus and adnexa and the immunohistochemical features, a primary carcinoma of the uterus, adnexa or peritoneum was considered unlikely. The presence of severe adenomyosis of the uterus, together with scattered extragenital endometriotic foci adjacent to the tumor, supported the diagnosis of an endometriosis-associated malignancy (see Fig. 9). This led to the conclusion that the malignancy had arisen from the previously diagnosed endometriosis.

The interdisciplinary tumor board recommended systemic therapy with carboplatin and paclitaxel, followed by radiotherapy of the thigh and, if necessary, the mons pubis. However, the initiation of prompt adjuvant therapy was rendered unfeasible due to delayed wound healing, a consequence of the extensive resection and utilisation of foreign material during the surgery.

Four months after surgery, an abdominal MRI scan revealed a recurrence of the disease, with multicystic formations in the right inguinal region extending to the mons pubis, the vulvar region and the proximal medial thigh. Furthermore, two new focal cystic lesions were identified supraumbilically with intra-abdominal extension. Postoperative lymph nodes in the right inguinal and inguinal region exhibited persistence and enlargement, while there was no evidence of pulmonary, visceral or osseous metastases. Following three cycles of carboplatin/paclitaxel chemotherapy, a subsequent CT scan of the thorax and abdomen revealed a size-progressive, polycystic relapse in the right inguinal region that traversed the midline, accompanied by increasing walling of the right common femoral artery and vein. Furthermore, a size-progressive, suspected metastatic lymph node was identified in the left inguinal region, while the lymph nodes in the right inguinal region, right iliac region and retroperitoneal region remained unchanged. Consequently, bevacizumab was added to the therapeutic regimen.

Following a total of six cycles of carboplatin/paclitaxel and three cycles of bevacizumab, a chest and abdominal CT scan revealed stable thoracoabdominal lymph nodes and a regressive cystic tumor mass in the right inguinal region. No evidence of pulmonary, visceral or osseous metastases was found. Following the administration of chemotherapy, the inguinal area was subjected to irradiation. Maintenance therapy with bevacizumab is currently ongoing. At the follow-up examination, 20 months after the procedure, the patient was in good general health.

## Discussion

Endometriosis is one of the most common benign gynecological conditions affecting women of reproductive age. However, its manifestation in surgical scars, particularly following abdominal procedures, is extremely rare, with an incidence ranging from 0.03 to 1.08% [37]. Malignant transformation of abdominal wall endometriosis into clear cell carcinoma is exceptionally rare, with a prevalence of 0.7-1.0% [30]. Clear cell carcinomas associated with endometriosis are considered a highly aggressive tumor, with a pronounced tendency to recur and metastasize. Due to the rarity of reported cases and the heterogeneity of the documentation regarding therapy, including its timing, duration, and outcomes, an evaluation of the therapy’s efficacy has not yet been conducted. The limited available evidence suggests a generally poor prognosis, with recurrence-free survival ranging from three to 93 months and a 5-year survival rate of approximately 40% [2, 29, 34]. In all documented cases the patient had previously undergone surgery on the internal genital organs, in particular caesarean section, as was also the case in our patient [3–5, 7, 8, 12, 13, 17, 18, 20, 21, 26, 29, 31, 32, 34, 36, 37, 40, 41, 48, 49]. Less frequently, other pervious interventions such as myoma enucleation [1], hysterectomy [23] or laparoscopic adnexae surgery [4, 29] had been documented. In many cases, endometriosis had not been previously diagnosed and symptoms had been absent. However, in some cases, endometriosis was known and had been treated according to guidelines [3, 5, 7, 20, 33, 34, 40]. An overview of the studies conducted is presented in Table 1.

**Table 1:** Comparative overview of relevant studies identified in the literature

	Year published	Age	Number of caesarean sections, (years ago)	Other previous operations (years ago)	Resection of the scar tumor, (years ago)	Previous diagnosis of endometriosis?	Symptoms since	Clinical findings (cm)	Tumor Marker CA125: U/ml	Tumor marker other: U/ml	Punch/open Biopsy, histo	Primary carcinoma operation	Mesh reconstruction	Histology	Systemic therapy	Recurrence	Recurerence treatment	Last info
Achach T [1]	2008	49	0	1Xfibroid enucleation (20y)		NA	Painful abd. wall mass	8,5	NA		No	TR	No	CCC, Endo	CHT recommended (patient refused it)	6 m local	3xcisplatin-C TR RT	NA
Alberto VO [3]	2006	38	1 (11y)	1x LT-ER (8y), 1x HE BSO (7y)		Yes	Abd. wall swelling (11y) increase in size (6 m)	5 × 4	NA		No	TR	Yes	CCC	6xCarb-Pac RT	NA	NA	NA
Bahall V 1 [4]	2022	46	0	1x LT- cyst (10y)		Yes	Enlarging abd. wall mass and pain (4 m)	10 × 8	1200	CEA: 10.2; CA15-3: 425.8; CA19-9: 6610.8	Yes (AC)	TR, HE, BSO, sampling P-LND	Yes	CCC	6xCarb-Pac	No		32 m
Bahall V 2 [4]	2022	57	2 (29, 27y)	2x umbilical hernia (26y), abd. HE (15y)		Yes	Enlarging abd. wall mass (1y); pain (4d)	15 × 15	NA		Yes (AC)	TR, BSO, P-LND	Yes	CCC, Endo, LN involvement	6xCarb-Pac	4 m I-P-LN	Re-I-P-LND Carb-Pac	NA
Bats AS [5]	2008	38	1 (13y)	0	1 (12y)	Yes	Enlarging abd. wall mass	10	NA		Yes (AC)	TR, HE, BSO, OE	Yes	CCC, Endo, adenomiosis	PST 3xCarb-Pac	8 m	Re-TR RT recommended (patient refused it)	NA
Behbehani S [7]	2019	48	1 (NA)	1xHE, ER (5y)		Yes	Enlarging abd. wall mass	7 × 5	NA		Yes (Endo)	TR, BSO, partial Bladder resection	No	CCC, Endo	2xLip-Dox-Gem	NA		NA
Bellalah A [8]	2022	45	2 (13, 11y)	0		No	Enlarging abd. wall mass (10y), pain, ulceration	20	NA		Yes (IC)	TR, HE, BSO	No	CCC	NA	No		12 m
Castagnino B [12]	2021	49	1 (20y)	0		No	Enlarging abd. wall mass (6 m), pain	11 × 11	49.8	CEA: 1.8; CA 19 − 9: 84	Yes (CCC)	TR, HE, BSO, I-P-LND	Yes	CCC, Endo, LN involvement	6xCarb-Pac-Bev	NA		NA
Colarossi C [13]	2021	52	2 (19, 17y)	0		No	Abd. wall mass	6.6 × 6.1	NA		Yes (IC)	TR	Yes	CCC	No	No		30 m
Feng Z 1 [17]	2022	48	1	NA	Yes	Yes	NA	NA	NA		NA	TR, HE, BSO, OE	NA	CCC	Carb-Pac	No		46 m
Feng Z 2 [17]	2022	44	1	NA	Yes	Yes	NA	NA	NA		NA	TR	NA	CCC	Carb-Pac	Progress, LK, local, pelvic metastasis		12 m dead
Feng Z 3 [17]	2022	49	1	NA	Yes	Yes	NA	NA	NA		NA	TR, HE, BSO, OE	NA	CCC	Carb-Pac	14 m local	TR	14 m
Feng Z 4 [17]	2022	46	1	NA	Yes	Yes	NA	NA	NA		NA	TR, HE, BSO, OE	NA	CCC	Carb-Pac	11 m local		18 m
Feng Z 5 [17]	2022	49	1	NA	Yes	Yes	NA	NA	NA		NA	TR	NA	CCC	Carb-Pac	22 m local	TR	24 m
Feng Z 6 [17]	2022	61	1	NA	Yes	Yes	NA	NA	NA		NA	TR, HE, BSO, OE	NA	CCC	Carb-Pac	10 m LK, local		59 m dead
Feng Z 7 [17]	2022	59	1	NA	Yes	Yes	NA	NA	NA		NA	TR, I-LND	NA	CCC, LN involvement	Carb-Pac	9 m LK	CHT	47 m
Feng Z 8 [17]	2022	42	1	NA	Yes	Yes	NA	NA	NA		NA	TR, HE, BSO, P-LND	NA	CCC, LN involvement	Carb-Pac	6 m LK	CHT	13 m
Feng Z 9 [17]	2022	42	1	NA	Yes	Yes	NA	NA	NA		NA	TR	NA	CCC	RT	3 m local	CHT	34 m
Feng Z 10 [17]	2022	39	1	NA	Yes	Yes	NA	NA	NA		NA	TR, HE, BSO, P-LND	NA	CCC	Carb-Pac	15 m local	CHT	16 m
Ferrandina G [18]	2016	44	1 (9y)	NA		No	Enlarging abd. wall mass (5 m),	22	NA	CEA: N; SSA: N	Yes (AC)	TR, HE, BSO, I-P-LND	Yes + fasciocutaneous flap	CCC, Endo, LN involvement	PST 3xCarb-Pac	2 m Liver	3xLip-Dox	4.5 m dead
Gentile JKA [20]	2018	42	1 (7y)	0	1 (2y)	Yes	Enlarging abd. wall mass (8 m)	10.6 × 8.3	NA	CEA: N; CA19-9: N; CA: N; AFP: N	NA	TR, I-P-LND	Yes	CCC, 1/8 LN involvemet	CHT recommended (patient refused it)	No		8 m
Giannella L [21]	2020	45	2 (22, 13y)	0		NA	Abd. wall mass, pain (3 m)	20	NA		Yes (Endo)	No	No	CCC	PST 3xCarb-Pac	Progress	MMetastasis RT Dox-Gem RT	7 m dead
Harris CM [23]	2024	46	0	HE (6y)		Yes	Abd. wall mass, pain (2w)	5.1 × 4.8	70	CA19-9: 47.3	Yes (CCC)	TR, HE, BSO, appendectomy, omentum-B	No	CCC	3xCarb-Pac-Bev (further treatment was refused) RT	No		6 m
Kadam S [26]	2021	55	2 (30y)	0		NA	Abd. wall mass, pain (9 m); rapid increase (2 m)	20 × 16	NA		No	TR, HE, BSO, OE, Sampling-LND	Yes + double-layer mesh inlay procedure	CCC	6xCarb-Pac	No		24 m
Lai YL 1 [29]	2019	52	2 (19y)	NA		NA	Enlarging abd. wall mass, ulceration	17.5	20.1		NA	TR, HE, BSO	NA	CCC, Endo, adenomiosis	No	10 m, I-LK, bone	No	14 m dead
Lai YL 2 [29]	2019	56	2 (33y)	NA		NA	Enlarging abd. wall mass	6.5	22.3		NA	TR, HE, BSO, OE, P-LND	NA	CCC	8xCarb-Pac	3 m, I-LK	I-LND	11 m dead
Lai YL 3 [29]	2019	52	0	1xSO (4y)		Yes	Enlarging abd. wall mass	7	38.5		NA	TR, HE, BSO	NA	CCC, Endo, adenomiosis	6xCarb-Pac	No		97 m
Lai YL 4 [29]	2019	56	1 (21y)			NA	Enlarging abd. wall mass	12	23		NA	TR, HE, BSO, P-LND	N/A	CCC	Carb-Pac	NA		NA
Lai YL 5 [29]	2019	55	3 (24y)			NA	Enlarging abd. wall mass	12.5	26.7		NA	TR, HE, BSO, OE, no R0 Resection	NA	CCC, Endo, adenomiosis	PST 5xCarb-Pac-Bev + 1xGem-Carb, adjuvant 3xGem-Carb-Bev	Progress		23 m dead
Lai YL 6 [29]	2019	45	3 (20y)			NA	Enlarging abd. wall mass, ulceration	4.8	38.9		Yes (CCC)	No		CCC	7xCarb-Pac + 1xLip-Dox-Carb RT	Progress		7 m dead
Li JY [31]	2003	38	1 (15y)	0		1	Intermittent pain (10y)	6 × 4	NA		Yes (AC)	TR, HE, BSO	No	CCC	3xEC-cisplatin	No		14 m
Liu H [33]	2014	39	1 (20y)		1 (15)	NA	Enlarging abd. wall mass, cyclical pain (10y)	6 × 5	22.1		No	TR, HE, BSO, I-P-paraaortal-LND, partial Bladder resection, Rx	NA	CCC, Endo, LN involvement	3xCarb-Pac (further treatment was refused) TCM	13 m		15 m dead
Lopes A [34]	2019	48	1 (18y)		1 (7y)	1	Rapid increase abd. wall mass (3 m)	11 × 9	3157.9		Yes (CCC)	TR, HE, BSO, OE, P-paraaortal-LND	Yes	CCC	6xCarb-Pac	No		6 m
Marques C [36]	2017	47	3 (17, 22, 24y)			N/A	Pain, increase abd. wall mass (3 m)	11	29		No	LT: BSO, TR; relapse (9 m): TR, HE	No/yes	CCC	6xCarb-Pac	9 m local	TR, 6xCarbo/Pac	36 m
Mihailovici A [37]	2017	47	1 (22)			NA	Pain, increase abd. wall mass (1y)	11.2 × 11	96		Yes (CCC)	TR, HE, BSO, OE	Yyes	CCC	9xCarb-Pac RT	NA		NA
Rivera Rolon M [40]	2019	48	3 (20,.y)	HE, ER	1 (20y)	1	Abd. wall swelling (1y); pain, increase mass (1 m)	7 × 5	NA		NA	TR	NA	CCC	CHT recommended (patient refused it)	NA		NA
Ruiz MP 1 [41]	2015	41	1 (20y)	0		NA	Pain, increase abd. wall mass (1y)	14.8 × 10.7	22		Yes (CCC)	TR, HE, BSO, partial OE	No	CCC, adenomiosis	6xCarb-Pac	6 m local	RT	NA
Ruiz MP 2 [41]	2015	57	3 (30y)	0		NA	Pain (9 m)	7	81	CEA: 96.5; CA19-9: N	Yes (CCC + Endo)	TR, HE, BSO, I-P-LND, Rx	Yes	CCC, Endo, LN involvement	3xCarb-Pac RT 3xCarb-Pac	No		NA
Schnieber D [43]	1986	40	1 (15y)	Appenektomie (19y), 1xAbort	No	No	Abd. wall mass (2y)	6	NA		NA	TR, HE, BSO, I-LNE	NA	CCC	High-dose gestagens, RT	12 m local		18 m dead
Stevens EE [45]	2013	51	G3/P2			NA	Increase abd. wall mass	NA	NA		Yes (CCC)	?		CCC	PST 3xCarb-Pac Op RT	No		6 m
Williams C [48]	2009	53	1 (17y)	0		0	Pain; increase abd. wall mass (4 m)	5 × 4	39		Yes (CCC)	TR, HE, BSO, OE, I-P-LND	No	CCC, adenomiosis, LN involvement	6x Carb-Pac	3 m		11 m dead
Womack H [49]	2022	37	NA	NA		NA	Pain	5 × 5	NA		Yes (CCC)	TR, HE, BSO	No	CCC	5xCarb-Pac RT	No		48 m
Current study		53	1 (19y)			0	Increase abd. wall mass (1y)	21 × 16	17.4		Yes (IC)	TR, HE, BSO, Sampling I-LND, partial Bladder resection, appendectomy, cholecystectomy	Yes + double-layer mesh inlay procedure	CCC, endometriosis, adenomios,	6xCarb-Pac- Bev RT Bev	4 m I-LK		20 m

*Abd *Abdominal, *BSO* Bilateral salpingo-oophorectomy, *B* Biopsy, *Bev* Bevacicumab, C Cyclophosphamide, *Carb* Carboplatin, *CCC* Clear cell carcinoma, *CHT* Chemotherapy, *d* day *E* Epirubicin, *Endo* Endometriosis, *ER* Endometriosis resection, *Gem*Gemcitabine, *HE* Hysterectomy, *IC* Inconclusive, *Lip*-*Dox* Liposomalem Doxorubicin, *LN* Lymph node, *LT* Laparoscopy treatment, *m* month, *N* Norm, *NA* Not available, *NED* No evidence of disease, *OE* Omentectomy, *Pac* Paclitaxel, *I*-*P*-*LND* Pelvic-lymph node dissection, *PST* Primary systemic treatment, *RT* Radiotherapy, *Rx* Margin-forming resection, *TCM* Traditional Chinese herbal medicine, *TR* Tumor resection, *w* week, *y* year

The majority of patients were approximately 50 years of age (with an age range from 37 [49] to 61 [17] years) at the time of diagnosis. The clinical spectrum ranged from silent abdominal-wall masses in women without a history of endometriosis to symptomatic lesions in patients with long-standing endometriosis. In many cases, asymptomatic swellings in the abdominal wall were observed over a period of months or years [3, 4, 31], before developing into rapidly growing, painful, nodular structures ranging from 5 [3] to 22 [18] cm in size. Scar endometriosis was sometimes diagnosed following the removal of a presumed scar tumor [20, 33, 34, 40]. In premenopausal patients, the disease often presented as a menstrual cycle-dependent, progressive nodule formation in the abdominal wall, associated with pain, while postmenopausal patients usually reported a painful, progressive nodule formation with occasional ulcerations [29, 41]. Observation of tumor growth often did not occur until years after the initial, mostly unrelated, abdominal surgical procedure, with documented intervals ranging from four years [29] to 27 years [4], and the lesions were often described to have infiltrated the entire abdominal wall. The present study demonstrates a high degree of congruence with the aforementioned criteria. Preoperative diagnosis is essential for planning further treatment. Conventional imaging techniques have proven unreliable in detecting malignancy, and the identification of specific markers remains elusive [15, 34]. Conventional tumor markers such as CEA, CA125, and CA19-9 were within the normal range in most reported cases, as observed in our patient [3, 13, 18, 20, 29, 33, 36, 48], however, some studies noted slightly increased values [12, 23, 37, 41] and a few even reported significantly increased values [4, 34]. Histopathological examination following fine-needle biopsy or open tissue sampling has confirmed an accurate diagnosis in several cases [4, 5, 12, 23, 31, 37]. However, due to tumor heterogeneity and size, achieving representative sampling is often difficult. Several reports have described biopsy specimens showing only endometriosis or other benign tissue [7, 8, 13, 21], with the correct diagnosis only being made on final histology. In our case, initial histopathological evaluation of the tumor, measuring over 12 cm and resected one year prior, revealed only endosalpingiosis. Fine-needle aspiration cytology yielded inconclusive results, and open biopsy demonstrated only endometriosis, providing no further diagnostic insight. Even intraoperative frozen-section analysis indicated endosalpingiosis exclusively. Therefore, the definitive diagnosis of clear cell carcinoma was established only upon final histopathological examination, highlighting the significant diagnostic challenges posed by these rare tumors.

The extant literature on the treatment of these patients is inconsistent, and there are currently no evidence-based guidelines for surgical or systemic management strategies [23]. Nevertheless, complete tumor resection is widely regarded as a crucial prognostic factor. These tumors are often very extensive at the time of diagnosis, so that radical excision with adjuvant therapy is usually required. The repair of resulting abdominal wall defects can pose a considerable surgical challenge, especially given that abdominal wall hernias occur in 12–50% of patients [15, 34]. In addition to tumor resection [1, 3, 7, 13, 17], other interventions such as hysterectomies with adnexectomy [4, 8, 17, 29, 49] and omentectomies [5, 17, 26, 29, 31, 33, 34, 37, 48] were frequently described. In addition, inguinal and/or pelvic lymphadenectomy [4, 12, 17, 18, 20, 26, 29, 33, 34, 41, 48] and para-aortic lymphadenectomy [33, 34] were also performed in some cases. Although no omental infiltration was detected in any of the cases, lymph node infiltration was observed in some of the patients who underwent lymphadenectomy [4, 12, 17, 18, 20, 33, 41, 48]. The management of defects in the abdominal wall depended on their extent, with primary closure or mesh reconstruction being the preferred approach [3–5, 12, 13, 18, 20, 36, 37, 41]. Ferrardine et al. described a reconstruction of the large abdominal wall defect using a Gore-Tex mesh and fasciocutaneous flaps [18], while Kadam et al. reported the successful application of a double mesh in an inlay procedure [26]. In the present case, the complete tumor removal procedure was performed in accordance with the extant literature. The surgical intervention involved complete excision resection, necessitating the removal of over 50% of the anterior abdominal wall, extending down to the pelvic bone, and encompassing the right inguinal region and a partial cystectomy. Concurrently, total abdominal hysterectomy with bilateral salpingo-oophorectomy was performed, with selective excision of enlarged inguinal lymph nodes, as described by numerous authors. The reconstruction of the extensive abdominal wall defect represented the most significant challenge, as only minimal residual fascia and muscle tissue remained. A single-stage reconstruction technique was chosen, which is employed only in exceptional cases at our institution and is scarcely reported in the literature. Macroscopically unremarkable omentum was positioned as a biological barrier between the intestine and a two-layer mesh to optimise postoperative function. The mesh was anchored to the pelvic bone using bone fixation anchors (see Fig. 6).

In the majority of cases, a chemotherapy regimen adapted from the standard protocol for clear cell ovarian cancer was utilized, consisting of carboplatin and paclitaxel, with or without the addition of bevacizumab [7]. In some cases, other chemotherapeutic agents such as doxorubicin and gemcitabine were administered [29]. The treatment with radiotherapy was documented in a few cases, either as monotherapy [7] or following chemotherapy [24]. In accordance with the extant literature, the patient received a chemotherapy regimen comprising carboplatin and paclitaxel only after complete wound healing had occurred and following an interdisciplinary case presentation to the multidisciplinary tumor board. Most recurrences are described in the literature as local or in the inguinal lymph nodes, which is how they presented in our patient upon follow-up CT. These were then treated with radiotherapy.

According to the results of modern genetic research, clear cell carcinomas show significant genetic differences compared to high-grade serous carcinomas. Specifically, mutations in the ARID1A and PIK3CA genes were identified with a higher frequency in clear cell carcinomas [19, 22]. It was found that the genes (ARID1A, PIK3CA, ATM, CHD4 and NRAS) in question also demonstrate a high mutation rate in endometriosis. This finding suggests the presence of a genetic correlation between endometriosis and clear cell carcinoma [46]. This hypothesis is further substantiated by the observation that identical mutations have been identified in both a clear cell ovarian carcinoma and the surrounding endometriosis tissue of a single patient [46]. In contrast, TP53 gene mutations, which are primarily responsible for high-grade serous ovarian carcinomas, occur less frequently in clear cell carcinomas [19, 46]. The present case study found that immunohistochemical analysis revealed a p53 wild-type mutation, L1CAM negativity and no microsatellite instability. These findings have the potential to pave the way for targeted therapies [19].

Clear cell carcinoma of the abdominal wall is an extremely rare condition, with the extant literature primarily consisting of isolated case reports or small case series. Consequently, there are currently no established diagnostic or therapeutic standards. A notable aspect addressed in the literature is the malignant transformation of scar endometriosis, especially after caesarean sections or other surgery of internal genital organs. It is imperative to acknowledge that endometriosis implants resulting from these procedures can be potential foci of malignancy. These implants often remain asymptomatic for extended periods, often spanning decades, until complications arise, underscoring the necessity for meticulous monitoring of patients with palpable tumor masses in the abdominal wall following such surgical interventions.

In cases of rapidly growing tumor masses within a scar following previous gynecological surgery, complete resection should be performed, even if the preoperative diagnostic measures, ranging from tumor marker determination and imaging diagnostics to histological investigations such as core biopsy or frozen section examination, are unremarkable. The definitive diagnosis of clear cell carcinoma can only be made during the final histological examination. Consequently, a radical approach is imperative to ensure complete resection, which is pivotal for prognosis. The preoperative diagnostic uncertainties may be attributable to the considerable size and inhomogeneity of the tumor, which can render it challenging to obtain a representative sample, and there is typically no increase in tumor markers. In addition to complete resection, particular attention should be paid to the careful selection of reconstruction techniques. In cases of large defects, mesh-based abdominal wall reconstruction, possibly with omentum plication, is an option for achieving optimal oncological and functional results. In view of the extant literature and the particulars of our case, the recurrent nature of the inguinal lymph nodes gives rise to the question of whether inguinal lymphadenectomy should be performed.

In the context of the high aggressiveness of the carcinoma, the high recurrence rate and the potential for metastasis, multimodal treatment with adjuvant chemotherapy and radiotherapy should be considered. Further studies and registries are required to refine these multimodal strategies, to identify effective combination therapies and to develop personalized treatment protocols. Furthermore, future studies should aim to optimize surgical procedures as much as possible to prevent the unintended spread of endometrial cells during gynaecological surgery.

Summary.

It can be concluded that gynecological surgeries, especially caesarean sections, are an important risk factor for the development of clear cell abdominal wall cancer where endometriosis is present. The transformation of these cells can occur over a period of decades, emphasising the necessity for prolonged and meticulous monitoring of affected patients. The rarity of this tumor entity, coupled with the paucity of data, results in the absence of standardized treatment recommendations.

In cases of rapidly growing abdominal wall tumors following previous gynecological surgery, radical resection with clean margins should be attempted, even in cases where a biopsy shows no evidence of cancer. In addition to complete tumor removal, the careful selection of suitable reconstruction procedures and, if necessary, a two-layer mesh reconstruction are crucial as effective methods of treating extensive defects to achieve optimal oncological and functional results. In addition to the surgical procedure, adjuvant therapies such as chemotherapy and radiotherapy play an important role in a multimodal treatment plan to further improve the prognosis, particularly due to the high aggressiveness and metastatic tendency of clear cell carcinoma.

The case described illustrates the challenges discussed in the literature and underlines the need for further research to improve the diagnosis and treatment of this very aggressive tumor. The development of targeted treatment options could help to improve the prognosis of this rare form of cancer.

## Data Availability

No datasets were generated or analysed during the current study.
